# Individualized ASO therapy for rare diseases

**DOI:** 10.1038/s43856-023-00255-3

**Published:** 2023-02-28

## Abstract

Annemieke Aartsma-Rus is a Professor of Translational Genetics at the Leiden University Medical Center. She was one of the pioneers of antisense oligonucleotide (ASO)-mediated exon skipping therapy for Duchenne muscular dystrophy (DMD). Her work focuses on the personalized approach to delivering exon skipping therapy for patients with rare genetic diseases within the setting of the Dutch Center for RNA Therapeutics, which she co-founded in 2020 and is on the Board of Directors. Currently, four exon skipping drugs have been approved by the US Food and Drug Administration for DMD, three of which are based on the IP generated by Aartsma-Rus’ institute. She is also involved in patient education in collaboration with the Duchenne Patient Academy and the European Organisation for Rare Diseases (Eurordis). In this Q&A, we ask Prof. Aartsma-Rus a series of questions on the latest developments in therapies for rare diseases and how best to overcome some of the existing challenges with this endeavour.

**Figure:**
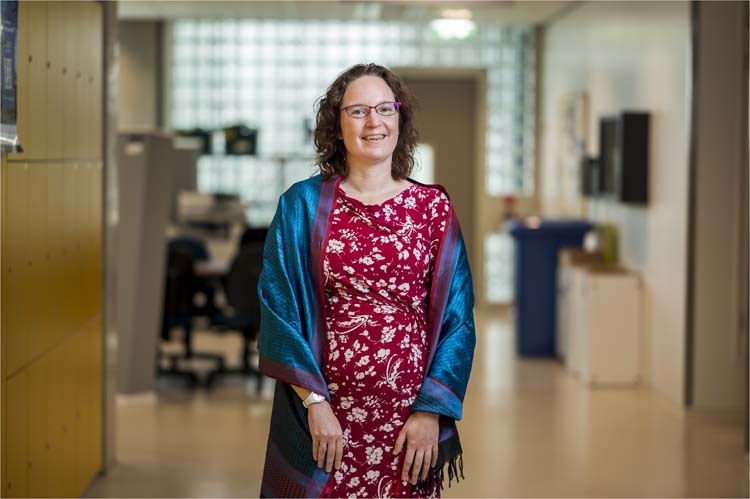
Property of Prinses Beatrix Spierfonds. All rights reserved.

How does your work contribute to the development of therapeutics for rare genetic diseases?

I have worked on developing ASO-mediated exon skipping therapy for DMD since 2000. This was one of the first ASO exon skipping therapies developed and tested in human clinical trials for DMD. This achievement involved scientific research, in addition to coordinating multi-lateral stakeholder education sessions between patient advocates, academics, as well as regulators under the auspices of the TREAT-NMD network. This network is composed of academics and patients, with the aim of promoting best practices for the management and treatment of neuromuscular disease patients, ensuring preparedness and infrastructure for trials towards future therapies.

Currently, I am applying the expertize gained from the DMD experience to develop exon skipping ASOs for eligible patients with progressive brain diseases. This work involves multi-faceted networks that run at a national level through the Dutch Centre for RNA Therapeutics, and another at the international level via the European 1 Mutation 1 Medicine (1M1M) and the global *N* = 1 collaborative (N1C). The purpose of these multi-layered networks is to align efforts on personalized treatments with ASOs. There are procedural differences at a national level, as well as between Europe and the USA, which is why alignment is needed at different levels. However, many of the processes and procedures will be the same across the globe, so whichever network develops something new it then gets shared within the wider community.

What are the most exciting advances pushing the boundaries of rare disease therapeutics?

The development of individualized exon skipping therapies. This is pioneering work as it has not been done in Europe and was done only for a few cases in the USA. The work is done in a very collaborative fashion, where we share successes and failures, and produce processes and procedures to streamline all efforts, as part of the N1C.

The challenges of developing individualized therapy are many, as the regular drug development path is not fit for purpose for *N* = 1 or *N* = few trials (i.e. the majority of rare diseases). Additionally, not only the therapy itself is individualized, but other aspects of the development are individualized, including outcome measures to test safety and efficacy. What is very appealing about this approach to me is that it is done in close collaboration with patients and families, as they are best suited to indicate what is relevant to them. Furthermore, it is very rewarding to work with a dedicated group that is focused on making *N* = 1 and *N* = few treatments a possibility for patients with unmet needs.

What are the main challenges in developing clinically applicable therapeutics for rare diseases?

In general, the biggest challenge is that there is no track record for clinical development for a given rare disease. This makes trial design and selecting optimal outcome measures to assess clinical benefit very difficult. Developing these outcome measures and collecting natural history takes time and money. Furthermore, innovative therapeutic approaches are used often, for which there is no proven track record. That means there is more uncertainty about the long-term efficacy and risks of these therapies. At the same time, rare disease therapy development is about as expensive as common disease therapy, but eligibility is restricted to a small number of patients. That means the cost of treatment per patient goes up due to increased manufacturing costs, as is the case for most innovative treatments (i.e. gene and cell therapy).

What are the existing barriers to delivering novel therapies to patients and how would they be best overcome?

The cost of treatment development in relation to the rarity of the disease. For the common rare diseases, it was possible to do clinical trials on hundreds of patients. However, the majority of rare diseases are ultra-rare, and the same rules do not apply. Hence, we need to find other ways to develop therapies for these patients.

We all know that therapies for rare diseases are costly. Ideally, we move to a more transparent system for the very small patient groups, where a fair price is paid for therapy. I propose a disruptive option that challenges how clinical drug development for rare diseases is currently conducted and evaluated. I am not proposing to forego safety and efficacy studies, but we must focus on what is most critical. Regular drug development takes time because we want to ensure treatments are safe and effective. However, many rare diseases are progressive, and patients will lose function each year or even each month. Given the risk of not treating the disease (loss of function), patients and families are likely to take more risks with regard to the uncertainty of side effects. Compare it with an ambulance: in case of emergency—it is OK to run red lights and go over the speed limit, provided it does not crash. We need to find that balance also for very rare disease therapy development.

Are there any other questions you think are important that you would like to answer?

I think what will be crucial to make individualized treatment and treatments for very small groups a reality is to share data. We can only move forward if we are transparent about what works and what does not work, about successes and failures. This is something that underpins the N1C as the first order of business is to share data and experience. I believe this should apply to all rare disease research. As scientists, we are very used to focusing on what works, but in this space, we can learn more from what does not work or was not safe, provided we are open about this.

